# Isolated Adrenocorticotropic Hormone Deficiency Mimicking Systemic Sclerosis: A Diagnostic Challenge in Patients With Rheumatoid Symptoms

**DOI:** 10.7759/cureus.80073

**Published:** 2025-03-05

**Authors:** Yohei Fujita, Masahiro Hatazaki

**Affiliations:** 1 Department of Diabetes and Endocrinology, Osaka General Medical Center, Osaka, JPN

**Keywords:** corticosteroid therapy, isolated adrenocorticotropic hormone deficiency, rheumatoid symptoms, secondary hypoadrenocorticism, systemic sclerosis

## Abstract

Isolated adrenocorticotropic hormone (ACTH) deficiency (IAD) is characterized by selective impairment of ACTH secretion while other anterior pituitary hormones remain unaffected. It is more common in men in their fifties, with autoimmune mechanisms likely playing a major role. Symptoms include fatigue, weight loss, and appetite loss. Some IAD patients also experience rheumatoid symptoms, such as muscle pain and joint stiffness. A 74-year-old male patient with type 2 diabetes mellitus developed worsening symptoms, including impaired consciousness, fatigue, and edema. A month before hospitalization, he was diagnosed with primary hypothyroidism and started on levothyroxine. However, his symptoms worsened, with finger contractures and joint swelling, prompting referral to our hospital. On admission, he showed signs of general malaise and facial changes. His skin showed sclerosis without characteristic features of systemic sclerosis (SSc), such as Raynaud’s phenomenon or digital ulcers. His lab tests showed negative autoantibodies for collagen diseases, and the patient did not meet the criteria for SSc. Further investigation revealed hyponatremia, hypoglycemia, and low ACTH and cortisol levels, indicating anterior pituitary hormone deficiency. Imaging confirmed an intact pituitary gland and hypothalamus. The patient was diagnosed with IAD and began intravenous hydrocortisone, which improved his symptoms, including hyponatremia, hypoglycemia, and hypotension. His skin sclerosis and joint swelling also improved. Thyroid function normalized, and levothyroxine was discontinued. After physical therapy, the patient was discharged. The symptoms of IAD are primarily related to cortisol deficiency. It is often managed with hydrocortisone supplementation, which leads to rapid improvement of clinical symptoms. Autoimmune mechanisms, including the presence of anti-pituitary antibodies, are thought to play a significant role in its pathogenesis. However, this case lacked anti-pituitary antibodies and other typical causes such as opioid use or immune checkpoint inhibitors. Thyroid dysfunction can occur in IAD patients, as cortisol suppresses the thyrotropin-releasing hormone (TRH)-thyroid-stimulating hormone (TSH) axis. In this case, the patient's thyroid dysfunction was resolved after hydrocortisone therapy. Rheumatoid symptoms, such as joint pain and skin changes, can also be present in IAD, leading to misdiagnosis as systemic diseases like SSc. Hydrocortisone therapy successfully improved these symptoms, highlighting the need for adrenal function testing in patients with unexplained rheumatoid symptoms. Some cases of IAD can mimic autoimmune diseases like SSc, complicating diagnosis and delaying treatment. It is important to consider IAD in patients with unexplained joint and skin symptoms. Additionally, thyroid hormone therapy may unmask adrenal insufficiency, underscoring the importance of evaluating adrenal function before initiating treatment.

## Introduction

The anterior pituitary gland secretes multiple hormones. When various causes, such as tumors, inflammation, trauma, bleeding, ischemia, and necrosis, damage the pituitary gland or hypothalamus, humans develop hyposecretion of pituitary hormones (generalized hypopituitarism, partial hypopituitarism) [1]. Among these, isolated adrenocorticotropic hormone (ACTH) deficiency (IAD) is characterized by selective impairment of ACTH secretion. Isolated adrenocorticotropic hormone deficiency is more common in men, with the average age of onset being in the fifties. The cause of IAD is unknown, but there are cases in which it is accompanied by other autoimmune diseases or in which anti-pituitary antibodies are present [1-3]. Clinical symptoms of IAD include generalized fatigue, loss of appetite, weight loss, and impaired consciousness [4]. Some patients with IAD may experience rheumatoid symptoms such as muscle pain, arthralgia, joint stiffness, and flexion contractures [5-7]. We report a case of a patient referred to our hospital with suspected systemic sclerosis (SSc) due to joint pain and finger contractures, who was ultimately diagnosed with IAD based on endocrinological tests.

## Case presentation

A 74-year-old man with type 2 diabetes mellitus, diagnosed at age 57, had been visiting a local physician for routine care. About six months before hospitalization, he frequently experienced impaired consciousness. A blood test revealed hypoglycemia, leading to the discontinuation of oral hypoglycemic agents. The physician prescribed antihypertensive medication for low blood pressure, after which his episodes of impaired consciousness ceased. Concurrently, he developed lethargy, fatigue, and edema. One month before hospitalization, laboratory findings revealed elevated thyroid-stimulating hormone levels, leading to a diagnosis of primary hypothyroidism. He was started on levothyroxine (50 μg daily), after which impaired consciousness recurred. His fingers became thick and hard, and his joints swelled and contracted. Suspecting SSc, his local physician referred him to our hospital.

On admission, he was in poor general condition, with a blank facial expression and slurred speech. Vital signs included a temperature of 37.5°C, blood pressure of 131/78 mmHg, a pulse of 78 beats/minute (regular), and oxygen saturation (SpO₂) of 98% on room air. He had anorexia and dysphagia. No cardiac murmurs were detected, and breath sounds were clear bilaterally. The abdomen was soft without tenderness. He exhibited finger flexion contractures and skin sclerosis but no Raynaud's phenomenon. He had no rash, digital ulcers, fingertip necrosis, or telangiectasia. His elbows, shoulders, and knees had limited, painful motion. Lower extremity weakness impaired his ability to walk.

Laboratory tests (Table 1) showed negative autoantibodies for collagen diseases. A chest X-ray ruled out interstitial lung disease. Blood tests were negative for anti-topoisomerase I and anticentromere antibodies. According to the 2013 American College of Rheumatology/European League Against Rheumatism Classification Criteria for SSc, the patient did not meet the criteria, as he scored only four points for skin sclerosis of the fingers and two points for gastrointestinal dysfunction (esophageal motility abnormalities) [8].

**Table 1:** The patient's test findings at admission

Variables	Patient value	Reference ranges
Complete blood count		
White blood cell counts	8,900 /µL	3,300-8,600 /µL
Neutrophils	65.50%	25-72%
Eosinophil	9.00%	0-7.0%
Basophil	0.40%	0-1.0%
Monocytes	9.20%	4.0-7.0%
Lymphocytes	15.90%	30-45%
Red blood cell counts	3.89x10^6^ /µL	4.2-5.6x10^6^ /µL
Hemoglobin	10.9 g/dL	13-17 g/dL
Hematocrit	32.20%	39-52%
Platelet counts	9.9x10^4^ /µL	1.3-3.6x10^4^ /µL
Biochemistry		
Albumin	3.0 g/dL	3.8-5.3 g/dL
Aspartate aminotransferase	25 IU/L	13-33 IU/L
Alanine aminotransferase	12 IU/L	8-42 IU/L
Lactate dehydrogenase	206 IU/L	119-229 IU/L
Alkaline phosphatase	191 IU/L	115-359 IU/L
γ-glutamyltransferase	17 IU/L	10-47 IU/L
Creatine kinase	107 IU/L	62-287 IU/L
Total bilirubin	0.7 mg/dL	0.3-1.2 mg/dL
Blood urea nitrogen	10 mg/dL	8-22 mg/dL
Serum creatinine	0.41 mg/dL	0.60-1.10 mg/dL
Serum uric acid	1.2 mg/dL	2.6-6.4 mg/dL
Serum sodium	119 mEq/L	138-146 mEq/L
Serum potassium	4.9 mEq/L	3.6-4.9 mEq/L
Serum chloride	85 mEq/L	99-109 mEq/L
Serum calcium	7.7 mg/dL	8.7-10.3 mg/dL
C-reactive protein	3.39 mg/dL	< 0.40 mg/dL
Urinary sodium	122 mmol/L	110-250 mmol/L
Collagen disease-related tests		
Antinuclear antibodies	< 40	< 40
Anti-topoisomerase I antibody	< 5.0 index	< 5.0 index
Anti-centromere antibodies	< 5 index	< 5 index
Anti-double-stranded deoxyribonucleic acid antibody	< 7.0 IU/mL	< 7.0 IU/mL
Anti-ribonucleoprotein antibody	< 5.0 index	< 5.0 index
Anti-smith antibody	< 5.0 index	< 5.0 index
Anti-Sjogren syndrome-A antibody	< 5.0 index	< 5.0 index
Anti-Sjogren syndrome-B antibody	< 5.0 index	< 5.0 index
Anti-Jo-1 antibody	< 7 index	< 7 index
Proteinase 3 anti-neutrophil cytoplasmic antibody	< 1.0 U/mL	< 1.0 U/mL
Myeloperoxidase-anti-neutrophil cytoplasmic antibody	< 1.0 U/mL	< 1.0 U/mL

Hyponatremia and hypoglycemia were managed with intravenous fluids. Oral medications, including levothyroxine and vasopressors, were discontinued. Urinary sodium levels exceeded 20 mmol/L, suggesting euvolemic hyponatremia. Hypertonic saline improved his level of consciousness. Despite physiological stress, ACTH and cortisol levels remained low, suggesting anterior pituitary insufficiency. Brain computed tomography (CT) revealed atrophy but no pituitary abnormalities. Magnetic resonance imaging (MRI) confirmed an intact pituitary gland and hypothalamus. A trunk CT scan showed no adrenal abnormalities.

**Figure 1:**
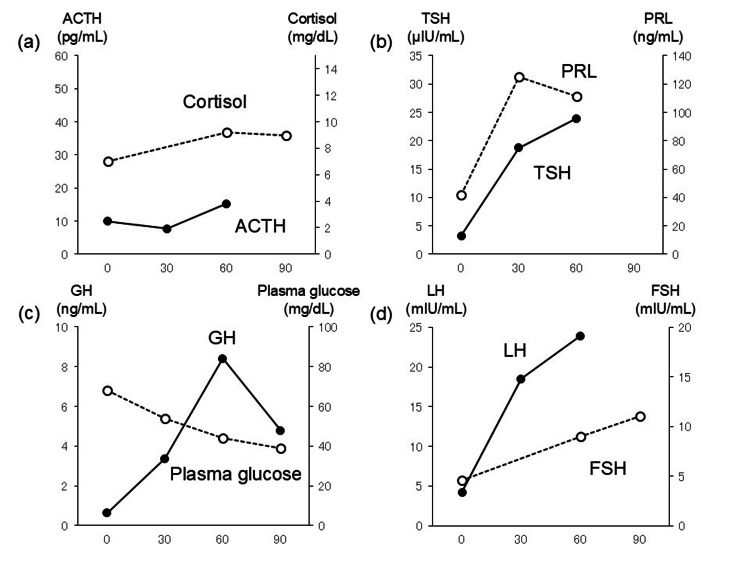
Anterior pituitary loading test (a) CRH test; (b) TRH test; (c) insulin hypoglycemia test; (d) LHRH test ACTH: adrenocorticotropic hormone; CRH: corticotropin-releasing hormone; TRH: thyrotropin-releasing hormone; TSH: thyroid-stimulating hormone; PRL: prolactin; LHRH: luteinizing hormone-releasing hormone; LH: luteinizing hormone; FSH: follicle-stimulating hormone; GH: growth hormone

Anterior pituitary function tests confirmed low ACTH and cortisol responses to CRH (Figure 1a). The patient had normal responses to thyrotropin-releasing hormone (TRH), luteinizing hormone-releasing hormone, and insulin hypoglycemia tests (Figure 1b, Figure 1c, and Figure 1d, respectively). He was diagnosed with IAD and started on intravenous hydrocortisone. Hyponatremia, hypoglycemia, and hypotension rapidly improved. Hydrocortisone was tapered to an oral maintenance dose of 10 mg in the morning and 5 mg in the evening [1,2]. Skin sclerosis and joint swelling improved with treatment. Since thyroid autoantibodies (anti-thyroglobulin and anti-thyroid peroxidase antibodies) were negative, Hashimoto's disease was unlikely. His thyroid function normalized after starting hydrocortisone, so we did not restart levothyroxine. During his illness, disuse syndrome progressed, leading to lower limb muscle weakness and dysphagia. Swallowing function improved with speech therapy, and the patient transitioned to a normal diet (1,600 kcal/day). After physical therapy, he was discharged on hospital day 72.

## Discussion

Symptoms of IAD include general fatigue, loss of appetite, impaired consciousness, weight loss, cognitive decline, and joint contractures [1,2,4,7]. In SSc, dysphagia is commonly observed due to fibrosis of the esophageal smooth muscle. Similarly, patients with IAD may develop dysphagia due to hyponatremia. In this case, the presence of dysphagia led to the suspicion of an esophageal motility disorder associated with SSc. However, many of these symptoms result from cortisol deficiency. Hydrocortisone, administered at a dose of 10-20 mg/day, is the standard treatment for IAD, leading to rapid improvement in symptoms such as hyponatremia, hypoglycemia, and hypotension [2].

In children, IAD is often caused by genetic mutations, such as those in the TBX19 (TPIT) or POMC genes, leading to congenital ACTH deficiency [9,10]. In adults, IAD can develop after trauma or lymphocytic hypophysitis, the latter of which may be due to autoimmune etiology [11-13]. There are reports that the use of opioids and immune checkpoint inhibitors may lead to the development of IAD [14-16]. In our case, anti-pituitary antibodies were negative, and pituitary cavities, seen in approximately one-third of cases of IAD, were not observed [3]. In addition, because he was not using opioids or immune checkpoints, the mechanism of IAD development in this case was unclear.

Cortisol supplementation often improves abnormal responses of other hormones. Since cortisol acts in an inhibitory manner on TRH-thyroid-stimulating hormone (TSH) secretion, high TSH levels are observed in some cases of IAD. According to a report by Murakami et al., thyroid dysfunction is often observed in patients with IAD. In more than 70% of such cases, the abnormalities of the pituitary-thyroid axis were temporary and were reversed by glucocorticoid replacement [17]. In our case, IAD was presumed to have become manifest when he was started on thyroid hormone replacement at a local hospital.

Hoshino et al. reported the case of a 61-year-old man with IAD who presented with diffuse muscle and joint pain [5]. The widespread musculoskeletal pain resolved with physiological doses of hydrocortisone replacement therapy. In our case, his symptoms, such as skin sclerosis and finger swelling, improved after starting steroid hormone therapy. In patients with rheumatoid symptoms of unknown etiology, adrenal insufficiency may be the cause. Initially, the symptoms suspected to be SSc were presumed to be due to IAD.

## Conclusions

Isolated adrenocorticotropic hormone deficiency can mimic autoimmune diseases like SSc, complicating diagnosis and delaying appropriate treatment. This case highlights the importance of considering endocrine disorders in patients with unexplained joint symptoms and skin changes. Additionally, thyroid hormone replacement therapy may unmask adrenal insufficiency, underscoring the necessity of adrenal function assessment before treatment initiation.
